# Supplementary material for: The encoding of touch by somatotopically aligned dorsal column subdivisions
Source: Nature. 2022 Nov 23;612(7939):310–5. doi: 10.1038/s41586-022-05470-x (PMC9729103; doi:10.1038/s41586-022-05470-x)
Supplement: Supplementary file 1 — Reporting Summary [file 41586_2022_5470_MOESM1_ESM.pdf]

nature portfolio

Corresponding author(s): David Ginty

Last updated by author(s): Oct 5, 2022

Reporting Summary

Nature Portfolio wishes to improve the reproducibility of the work that we publish. This form provides structure for consistency and transparency in reporting. For further information on Nature Portfolio policies, see our Editorial Policies and the Editorial Policy Checklist.

Statistics

For all statistical analyses, confirm that the following items are present in the figure legend, table legend, main text, or Methods section.

n/a	Confirmed	
☐	☒	The exact sample size (*n*) for each experimental group/condition, given as a discrete number and unit of measurement
☐	☒	A statement on whether measurements were taken from distinct samples or whether the same sample was measured repeatedly
☐	☒	The statistical test(s) used AND whether they are one- or two-sided<br>*Only common tests should be described solely by name; describe more complex techniques in the Methods section.*
☒	☐	A description of all covariates tested
☐	☒	A description of any assumptions or corrections, such as tests of normality and adjustment for multiple comparisons
☐	☒	A full description of the statistical parameters including central tendency (e.g. means) or other basic estimates (e.g. regression coefficient) AND variation (e.g. standard deviation) or associated estimates of uncertainty (e.g. confidence intervals)
☐	☒	For null hypothesis testing, the test statistic (e.g. *F*, *t*, *r*) with confidence intervals, effect sizes, degrees of freedom and *P* value noted<br>*Give P values as exact values whenever suitable.*
☒	☐	For Bayesian analysis, information on the choice of priors and Markov chain Monte Carlo settings
☒	☐	For hierarchical and complex designs, identification of the appropriate level for tests and full reporting of outcomes
☒	☐	Estimates of effect sizes (e.g. Cohen's *d*, Pearson's *r*), indicating how they were calculated

Our web collection on statistics for biologists contains articles on many of the points above.

Software and code

Policy information about availability of computer code

Data collection	Electrophysiology data was collected using pClamp 11 (juxtacellular recordings) or Intan RHX 3.0.4 (Multi-electrode array recordings).<br>Anatomical data was collected using Zen Blue or Zen Black (2012). Images of the paw and behavior were collected using Spinview 2.3.0.77.
Data analysis	Data were analyzed using custom scripts written in Matlab R2019a.<br>Custom scripts used in this study are posted on Github (see Methods for link).<br>Electrophysiology data collected using multielectrode arrays were analyzed using Kilosort 2.0 and Phy (Pachitaru et al. 2016; Rossant et al. 2016).<br>Figures were generated in Igor 6.37.

For manuscripts utilizing custom algorithms or software that are central to the research but not yet described in published literature, software must be made available to editors and reviewers. We strongly encourage code deposition in a community repository (e.g. GitHub). See the Nature Portfolio guidelines for submitting code & software for further information.

Data

Policy information about availability of data

All manuscripts must include a data availability statement. This statement should provide the following information, where applicable:

- Accession codes, unique identifiers, or web links for publicly available datasets
- A description of any restrictions on data availability
- For clinical datasets or third party data, please ensure that the statement adheres to our policy

Source data are available with the published paper. Datasets generated in this study are available from the corresponding author upon reasonable request.

Field-specific reporting

Please select the one below that is the best fit for your research. If you are not sure, read the appropriate sections before making your selection.

☒ Life sciences ☐ Behavioural & social sciences ☐ Ecological, evolutionary & environmental sciences

For a reference copy of the document with all sections, see nature.com/documents/nr-reporting-summary-flat.pdf

Life sciences study design

All studies must disclose on these points even when the disclosure is negative.

Sample size	Sample size was not predetermined. Sample sizes were based on previous studies from our lab and others that are common in the field (Choi et al. 2020; Lehnert et al. 2021; Petty et al. 2021).
Data exclusions	No data were excluded from analysis unless it failed to meet quality standards, except the following:<br>For experiments in Extended Data Figure 7, units that had initially high thresholds at baseline and became mechanically insensitive following DCN lesion were not included for analysis because stimuli may have been off center of the receptive field.
Replication	We performed experiments with multiple animals to confirm reproducibility. All attempts at replication were successful. The number of replications is noted in Extended Data Table 1.
Randomization	Most experiments in this study did not compare separate groups, so randomization was not used. For pharmacology experiments, mice were randomized to either receive drug application or not.
Blinding	It was not possible to perform blind experiments in this study as mice and treatments were easily identifiable as the experiments were performed.

Reporting for specific materials, systems and methods

We require information from authors about some types of materials, experimental systems and methods used in many studies. Here, indicate whether each material, system or method listed is relevant to your study. If you are not sure if a list item applies to your research, read the appropriate section before selecting a response.

Materials & experimental systems

n/a	Involved in the study	
☐	☒	Antibodies
☒	☐	Eukaryotic cell lines
☒	☐	Palaeontology and archaeology
☐	☒	Animals and other organisms
☒	☐	Human research participants
☒	☐	Clinical data
☒	☐	Dual use research of concern

Methods

n/a	Involved in the study	
☒	☐	ChIP-seq
☒	☐	Flow cytometry
☒	☐	MRI-based neuroimaging

Antibodies

Antibodies used	PRIMARIES:<br>mouse anti-NeuN, 1:1000 MAB377, Millipore, Clone A60<br>guinea pig anti-Vglut1, 1:2000 135301, Synaptic Systems<br>SECONDARIES:<br>goat anti-mouse Alexa-488, 1:500, ab150113, Abcam<br>IB4-Alexa 647, 1:300, I32450, ThermoFisher<br>FITC goat anti-GFP, 1:500, ab6662, Abcam
Validation	mouse anti-NeuN (1:1000, MAB377, Millipore): Abraira et al. Cell, 2017<br>guinea pig anti-Vglut1, (1:2000 135301, Synaptic Systems): Validated by vendor; performed western blot on brain homogenate of wildtype and vglut1 KO mice.

Animals and other organisms

Policy information about studies involving animals; ARRIVE guidelines recommended for reporting animal research

Laboratory animals	This study uses mice. Mice were older than postnatal day 40 and were of either sex.<br>The following mouse lines were used: C57Bl/J6; Calca-FlpE (Choi et al. Nature 2020); AvilFlpO (Choi et al. Nature 2020); AvilCre (Zhou et al. PNAS 2010); Cdx2-Cre (Coutaud & Pilon Genesis 2013); RosaLSL-Acr1 (Li et al. Elife 2019); RosaFSF-ReaChR (derived from Hooks

	et al. Journal of Neuroscience 2015); Animals were maintained on mixed C57Bl/J6 and 129S1/SvImJ backgrounds. C57Bl/J6 were obtained from Jackson Laboratories.
Wild animals	This study did not involve wild animals.
Field-collected samples	This study did not involve field-collected samples.
Ethics oversight	All experimental procedures were approved by the Harvard Medical School Institutional Care and Use Committee and were performed in compliance with the Guide for Animal Care and Use of Laboratory Animals.

Note that full information on the approval of the study protocol must also be provided in the manuscript.
